# Assessing response bias from missing quality of life data: The Heckman method

**DOI:** 10.1186/1477-7525-2-49

**Published:** 2004-09-16

**Authors:** Anne E Sales, Mary E Plomondon, David J Magid, John A Spertus, John S Rumsfeld

**Affiliations:** 1 HSR&D Center of Excellence, VA Puget Sound Health Care System, University of Washington, Seattle, WA, USA; 2 Department of Health Services, University of Washington, Seattle, WA, USA; 3 Cardiovascular Outcomes Research, Denver VA Medical Center, and Division of Cardiology, University of Colorado Health Sciences Center, Denver, CO, USA; 4 Clinical Research Unit, Colorado Permanente Medical Group, and Department of Preventive Medicine and Biometrics, University of Colorado Health Sciences Center, Denver, CO, USA; 5 Cardiovascular Outcomes Research, Mid-America Heart Institute, and Department of Medicine, University of Missouri, Kansas City, MO, USA

**Keywords:** Selection bias, cardiovascular disease, health-related quality of life

## Abstract

**Background:**

The objective of this study was to demonstrate the use of the Heckman two-step method to assess and correct for bias due to missing health related quality of life (HRQL) surveys in a clinical study of acute coronary syndrome (ACS) patients.

**Methods:**

We analyzed data from 2,733 veterans with a confirmed diagnosis of acute coronary syndromes (ACS), including either acute myocardial infarction or unstable angina. HRQL outcomes were assessed by the Short-Form 36 (SF-36) health status survey which was mailed to all patients who were alive 7 months following ACS discharge. We created multivariable models of 7-month post-ACS physical and mental health status using data only from the 1,660 survey respondents. Then, using the Heckman method, we modeled survey non-response and incorporated this into our initial models to assess and correct for potential bias. We used logistic and ordinary least squares regression to estimate the multivariable selection models.

**Results:**

We found that our model of 7-month mental health status was biased due to survey non-response, while the model for physical health status was not. A history of alcohol or substance abuse was no longer significantly associated with mental health status after controlling for bias due to non-response. Furthermore, the magnitude of the parameter estimates for several of the other predictor variables in the MCS model changed after accounting for bias due to survey non-response.

**Conclusion:**

Recognition and correction of bias due to survey non-response changed the factors that we concluded were associated with HRQL seven months following hospital admission for ACS as well as the magnitude of some associations. We conclude that the Heckman two-step method may be a valuable tool in the assessment and correction of selection bias in clinical studies of HRQL.

## Background

The potential impact of missing survey responses is often ignored in health-related quality of life (HRQL) studies [1,2]. Missing data from study participants can cause bias in parameter estimates of models predicting HRQL outcomes [2-4]. Unfortunately, regression models are frequently interpreted with the assumption that available data are representative of the entire study population. Researchers may compare clinical characteristics of respondents with and without missing surveys, but rarely attempt to assess the impact of these differences on the regression model parameter estimates and ultimately on the results of the study. The assumption that there are minimal or no effects on parameter estimates is only reasonable if one can demonstrate that data missing from a study are truly missing at random, making them ignorable, which is rarely the case [2,4].

Newer statistical techniques have been developed to assess and correct for bias resulting from missing HRQL surveys [2,3]. One technique which has received little attention in the medical literature to date is the Heckman two-step method [5-8]. The Heckman method was developed by an economist, James Heckman, to address problems of self-selection among women participating in the labor force. This method makes it possible to assess whether selection bias is present, identify factors contributing to the selection bias, and to control for this bias in estimating the outcomes of interest. The Heckman method attempts to control for the effect of non-random selection by incorporating both the observed and unobserved factors that affect non-response.

The objective of this study was to demonstrate the use of the Heckman two-step method to assess and correct for bias due to missing HRQL surveys. To accomplish this goal, we evaluated HRQL outcomes in a cohort of patients with acute coronary syndromes (ACS).

## Methods

### Study population

We analyzed data from the VA Access To Cardiology study, which was a multi-center prospective cohort study of 2,733 veterans with a confirmed diagnosis of acute coronary syndromes (ACS), including either acute myocardial infarction or unstable angina [9]. Baseline patient characteristics (demographic, cardiac history, non-cardiac history and hospitalization variables) were collected at the time of ACS hospitalization. HRQL outcomes were then assessed by the Short-Form 36 (SF-36) health status survey which was mailed to all patients who were alive 7 months following ACS discharge. A second mailed survey was sent to non-respondents. If no response was obtained from the mailed surveys, attempts were made to contact the patients by phone. Of the 2,733 patients in the study, 1,660 (61%) completed the survey, 306 (11%) died, and 767 (28%) were alive and did not complete the survey. Of those completing the survey, most responded to the first mailing with much smaller numbers responding to the second mailing or to phone calls.

### Variables

The outcome variables were the Physical Component Summary (PCS) and Mental Component Summary (MCS) scores from the 7-month SF-36 health status survey. The PCS and MCS scores reflect a patient's overall physical and mental health status, respectively [10,11]. The PCS and MCS scores are continuous variables with a range of 0–100, where higher scores indicate better health status. We constructed a dichotomous variable to indicate whether the patient responded to the SF-36 or not. It is important to note that models of HRQL outcomes may be biased both because subjects may have died before survey administration (survivor bias) or because of survey non-response in subjects that were alive at the time of survey administration [2]. However, the best way to handle patients who die prior to administration of the HRQL survey remains controversial [12]. Since the focus of this paper was to demonstrate the use of the Heckman model rather than methods of dealing with death in HRQL studies, we included only those patients who survived 7-months and were therefore eligible to complete the survey. Candidate predictor variables included a wide array of demographic, cardiac, and non-cardiac variables from the index hospitalization, and selected variables from the interim period between discharge and the 7-month SF-36 health status survey (Table 1). These variables were derived from the established literature on risk factors for adverse post-MI outcomes (mortality, functional status, and HRQL) [13-20]. Patient demographic and clinical data from the index hospitalization and 7-month follow-up period were abstracted from the electronic medical record and/or from national VA patient care databases.

**Table 1:** Comparison of SF-36 Survey Responders to Non-Responders

**Variables**	**SF 36 responders**	**SF 36 non-responders**	**P-value**
***Demographics***			

Mean patient age	65.5	62.8	<.01
Male gender	97.7%	97.5%	0.69
Caucasian race	78.4%	80.0%	0.21
***Prior Cardiac History***			

Hx of MI	38.0%	36.6%	0.33
Hx of Chronic heart failure	18.2%	18.3%	0.90
Hx of Coronary artery bypass graft surgery	26.3%	23.0%	0.01
Hx of Percutaneous coronary intervention	17.2%	12.6%	<.01
***Prior Non-Cardiac History***			

History of trauma	0.4%	0.7%	0.31
Substance or alcohol abuse	15.6%	21.0%	<.01
Current smoker	35.7%	43.0%	<.01
Dementia	2.0%	3.3%	<.01
COPD	29.7%	26.1%	0.01
Stroke	10.8%	13.2%	0.01
Depression	28.7%	40.5%	<.01
Arthritis	54.8%	54.4%	0.92
Diabetes mellitus	31.6%	32.0%	0.73
Peptic ulcer disease	11.1%	9.6%	0.26
Events during Index Admission and Interim Period*			

Coronary revascularization during index admission	26.6%	23.9%	0.05
Cardiogenic shock	1.1%	1.1%	0.94
Hypotensive episode	8.7%	9.2%	0.59
Do not resuscitate order	1.2%	2.9%	<.01
Positive stress test	12.1%	11.1%	0.32
Admitted to Tertiary VA	63.9%	69.5%	0.01
ST-segment elevation MI on ECG	19.0%	21.4%	0.05
Mean serum creatinine	1.17	1.19	0.58
Left bundle branch block on ECG	3.6%	3.2%	0.52
Cardiac catheterization during index admission	33.4%	31.4%	0.16
Discharge diagnosis Unstable Angina (vs. MI)	49.4%	46.5%	0.06

* Interim period is defined as the time period between discharge from index ACS hospitalization and the 7-month HRQL survey assessment.

MI: myocardial infarction; Hx: History; COPD: chronic obstructive pulmonary disease; ECG: electrocardiogram

### Analyses

Baseline characteristics of the patients who did and did not complete an SF-36 were compared using analysis of variance for continuous variables and chi-square for categorical variables (Table 1). Then, a series of regression models were developed, including 1) initial PCS and MCS models which did not account for potential bias due to missing surveys, 2) Heckman selection models (modeling response to the SF-36), and finally 3) final PCS and MCS models (accounting for potential bias due to missing surveys). We used robust regression for all equations (Stata version 8.0 SE), controlling for cluster sampling by VA medical center. In prior analyses, we established that the intra-class correlation, the measure of the effect of clustering by medical center in this case, was significant. As a result, it was necessary to control for bias due to autocorrelation, or similarity among patients within a medical center, compared to patients at a different medical center. Stata uses the Huber-White estimator to control for the bias due to clustering. This technique deflates the standard errors of the parameter estimates, in this case the coefficients, correcting the inference statistics.

### Overview of the Heckman method

There are two steps in the Heckman method. The first step is the development of a selection equation (i.e. a model of factors associated with survey non-response). This step includes derivation of a variable from the selection equation called the Inverse Mills Ratio (IMR). The second step of the Heckman method is the insertion of the IMR variable into the initial regression models (e.g. those not accounting for potential bias due to missing surveys) from a given study in order to assess for, and attempt to control for, selection bias.

### Heckman method: Step one

The first step in the Heckman method is to create the selection model, which estimates whether or not the quality of life survey was completed. The Heckman selection equation is usually estimated using a probit estimator [5,21]. The probit estimator requires a binary outcome variable, in this case whether the patient responded to the SF-36 or not (coded 1 for responder, 0 for non-responder). The candidate predictor variables for the selection model were those listed in Table 1. Although the Heckman selection equation will usually have multiple variables, some of which will be the same variables that enter the multivariable models of HRQL outcomes, it is important that the Heckman selection equation contain at least one variable that can legitimately be excluded from the initial models to safeguard against colinearity between the Heckman selection equation and the initial regression models. This means that this variable (or set of variables) is, in theory, a factor influencing whether someone responded to the questionnaire, but not a factor in predicting their component scores on the SF-36. This variable or set of variables is called an instrument in econometrics, and should be a strong predictor of response in the selection equation. We therefore stress that it is essential that the candidate variables considered for the Heckman selection equation be as comprehensive as possible, not omitting any variables that may contribute to whether a person responds to the survey.

Once the Heckman selection equation is estimated, the residuals (error term) from this equation are used to form a new variable called the Inverse Mills Ratio (IMR). The formula to create the IMR variable depends on the distributional assumption in the outcome equation. In most HRQL applications, the quality of life score is the outcome of interest and is usually estimated using multivariable linear regression. In this case, the distributional assumption of the error term is the standard normal distribution, so that the ratio of the standard normal probability density function (pdf) and cumulative density function (cdf) applied to the residuals for each individual in the data set is created. The ratio of pdf/cdf is the IMR.

Each individual in the study sample receives an individual value of the IMR based on the residual observed for that individual in the selection equation. In this study, the value of the IMR for each individual represented the predicted probability that they completed the 7-month SF-36 survey. It is important to note that the IMR is a function not only of observed or measured variables that are included in the selection equation, but also of unobserved or unmeasured variables. These are captured through the error term or residual in the selection equation, and included through the non-linear function used to estimate the IMR. As a result, adding the IMR into the outcome equation introduces a term that attempts to capture both observed and unobserved variables that affect selection, or non-response.

We estimated the Heckman model using the maximum likelihood estimation method in Stata version 8.0. In this approach, the outcome and selection models are estimated jointly, which can result in slightly different selection models for different outcomes, in this case the PCS and MCS scores from the SF-36. However, for clarity of presentation of the Heckman process, we present only one table of selection equation results (the probit estimation of the probability of returning the SF36), assuming that the patient survived to the 7-month survey point.

### Heckman method: Step two

The second step of the Heckman method is to include the IMR as a separate predictor variable in the initial regression models. In this study, the IMR variable derived from our Heckman selection model was inserted into the initial PCS and MCS models. Once this variable is inserted, two factors can be evaluated to help determine whether there is significant bias from missing responses in the initial models. First, one can examine the significance of the IMR variable itself. If significant, it suggests there was significant bias in the initial model. However, one potential limitation of the Heckman method is that if the Heckman selection model is not well-specified, and the variables in the selection model do not predict response/non-response well, the IMR may be weaker than expected and the Heckman method may have limited power to detect bias. Therefore, a second factor to examine following the addition of the IMR variable into the initial outcome models is whether or not there have been significant changes in any of the parameter estimates of the other predictor variables in the model. While somewhat arbitrary, changes in parameter estimates of >10% may indicate that these estimates were biased due to missing surveys. Where possible, one should apply clinical judgment about whether changes in parameter estimates are 'biologically important' [22].

With these factors taken together, the insertion of the IMR variable into the initial risk models allows the assessment of whether or not there was bias in the initial models, and suggests which initial predictors may have been most associated with this bias. Furthermore, by including the effect of unmeasured as well as measured variables from the selection equation, bias due to selection is controlled.

## Results

### Baseline characteristics

Compared to patients that completed the 7-month SF-36 survey, patients who did not respond to the survey were older, more likely to be current smokers and more likely to have a history of alcohol or substance abuse, dementia, stroke, or depression (Table 1). Furthermore, the non-responders were more likely to have had ST-segment elevation on their ECG, more likely to have been admitted to a tertiary care VA hospital, and were more likely to have had a do not resuscitate order during their index hospitalization. Survey non-responders were less likely to have a history of prior coronary artery bypass graft (CABG) surgery, prior percutaneous coronary intervention (PCI), or chronic obstructive pulmonary disease (COPD), and were less likely to receive coronary revascularization during index hospitalization.

### Initial PCS and MCS models

The initial multivariable PCS and MCS models not accounting for potential selection bias from missing HRQL surveys are presented in Tables 3 and 5.

**Table 2:** Heckman Selection Model (N = 1605)

		**95% Confidence Interval**	
**Variables**	**Coefficient**	**Lower Limit**	**Upper Limit**
Mean patient age	**0.01**	**0.00**	**0.02**
Male gender	0.44	-0.07	0.95
Caucasian race	-0.06	-0.25	0.13
Hx of MI	0.02	-0.16	0.20
Hx of Chronic heart failure	-0.01	-0.20	0.18
Hx of Coronary artery bypass graft surgery	0.09	-0.13	0.31
Hx of Percutaneous coronary intervention	**0.28**	**0.01**	**0.54**
History of trauma**	-0.29	-1.37	0.80
Substance or alcohol abuse	**-0.21**	**-0.40**	**-0.01**
Current smoker	-0.10	-0.29	0.09
Dementia	0.02	-0.76	0.79
COPD	**0.23**	**0.05**	**0.41**
Stroke	-0.12	-0.39	0.15
Depression	**-0.26**	**-0.48**	**-0.05**
Arthritis	0.08	-0.07	0.23
Diabetes mellitus	-0.02	-0.25	0.21
Peptic ulcer disease**	0.04	-0.20	0.28
Coronary revascularization during index admission	0.12	-0.06	0.30
Cardiogenic shock	0.04	-0.81	0.89
Hypotensive episode	0.05	-0.29	0.38
Do not resuscitate order**	**-0.68**	**-1.09**	**-0.27**
Positive stress test	-0.01	-0.25	0.24
Admitted to Tertiary VA	-0.09	-0.28	0.10
ST segment elevation MI on ECG	-0.04	-0.29	0.22
Mean serum creatinine	0.01	-0.06	0.07
Left bundle branch block on ECG	-0.02	-0.41	0.38
Cardiac catheterization during index admission	0.04	-0.10	0.19
Discharge diagnosis Unstable Angina (vs. MI)	0.06	-0.20	0.32

MI: myocardial infarction; Hx: History; COPD: chronic obstructive pulmonary disease; ECG: electrocardiogram;

**Instruments not included in outcomes equations

**Table 3:** Initial PCS Model

		**95% Confidence Interval**	
**Variables**	**Coefficient**	**Lower Limit**	**Upper Limit**
Mean patient age (per 1 year increment)	**-0.06**	**-0.11**	**-0.01**
Male gender	-1.04	-4.31	2.23
Caucasian race	-0.69	-1.72	0.35
Hx of MI	-0.85	-2.46	0.75
Hx of Chronic heart failure	**-2.96**	**-4.36**	**-1.55**
Hx of Coronary artery bypass graft surgery	**-3.12**	**-4.29**	**-1.95**
Hx of Percutaneous coronary intervention	-1.09	-2.68	0.51
Substance or alcohol abuse	-0.30	-1.89	1.30
Current smoker	-0.57	-1.66	0.52
Dementia	-0.84	-3.17	1.50
COPD	**-3.76**	**-4.77**	**-2.76**
Stroke	**-3.01**	**-4.34**	**-1.67**
Depression	**-4.61**	**-6.07**	**-3.15**
Arthritis	**-1.89**	**-2.94**	**-0.83**
Diabetes mellitus	**-2.33**	**-3.56**	**-1.09**
Coronary revascularization during index admission	**1.91**	**0.38**	**3.44**
Cardiogenic shock	1.62	-3.88	7.12
Hypotensive episode	0.13	-1.93	2.19
Positive stress test	1.15	-0.47	2.76
Admitted to Tertiary VA	-0.88	-2.00	0.24
ST-segment elevation MI on ECG	**2.34**	**0.81**	**3.86**
Mean serum creatinine (per 1 mg/dl increment)	**-0.64**	**-1.13**	**-0.16**
Left bundle branch block on ECG	0.09	-2.43	2.61
Cardiac catheterization during index admission	0.63	-0.45	1.71
Discharge diagnosis Unstable Angina (vs. MI)	-0.66	-1.54	0.21
Inverse Mills Ratio *			

* Inverse Mills Ratio: Variable derived from the Heckman Selection Equation; MI: myocardial infarction; Hx: History; COPD: chronic obstructive pulmonary disease; ECG: electrocardiogram

**Table 4:** PCS Model Corrected For Response Bias (N = 1605)

		**95% Confidence Interval**	
**Variables**	**Coefficient**	**Lower Limit**	**Upper Limit**
Mean patient age (per 1 year increment)	**-0.07**	**-0.11**	**-0.03**
Male gender	-1.00	-4.05	2.04
Caucasian race	-0.67	-1.62	0.28
Hx of MI	-0.89	-2.39	0.62
Hx of Chronic heart failure	**-2.95**	**-4.22**	**-1.69**
Hx of Coronary artery bypass graft surgery	**-3.16**	**-4.23**	**-2.09**
Hx of Percutaneous coronary intervention	-1.24	-2.75	0.28
Substance or alcohol abuse	-0.17	-1.85	1.52
Current smoker	-0.48	-1.48	0.51
Dementia	-0.77	-2.89	1.34
COPD	**-3.91**	**-4.91**	**-2.91**
Stroke	**-2.92**	**-4.14**	**-1.70**
Depression	**-4.68**	**-6.06**	**-3.31**
Arthritis	**-1.71**	**-2.75**	**-0.67**
Diabetes mellitus	**-2.33**	**-3.45**	**-1.20**
Coronary revascularization during index admission	**1.76**	**0.32**	**3.19**
Cardiogenic shock	1.44	-3.72	6.60
Hypotensive episode	0.48	-1.50	2.46
Positive stress test	1.06	-0.38	2.50
Admitted to Tertiary VA	-0.78	-1.81	0.25
ST-segment elevation MI on ECG	**2.41**	**0.97**	**3.85**
Mean serum creatinine (per 1 mg/dl increment)	**-0.66**	**-1.11**	**-0.22**
Left bundle branch block on ECG	0.05	-2.30	2.39
Cardiac catheterization during index admission	0.60	-0.41	1.60
Discharge diagnosis Unstable Angina (vs. MI)	-0.62	-1.42	0.19
Inverse Mills Ratio *	-2.15	-5.66	1.37

* Inverse Mills Ratio: Variable derived from the Heckman Selection Equation; MI: myocardial infarction; Hx: History; COPD: chronic obstructive pulmonary disease; ECG: electrocardiogram

**Table 5:** Initial MCS Model

		**95% Confidence Interval**	
**Variables**	**Coefficient**	**Lower Limit**	**Upper Limit**
Mean patient age (per 1 year increment)	**0.08**	**0.04**	**0.13**
Male gender	0.14	-3.07	3.36
Caucasian race	-0.69	-1.51	0.12
Hx of MI	**-1.19**	**-2.36**	**-0.02**
Hx of Chronic heart failure	0.01	-1.05	1.07
Hx of Coronary artery bypass graft surgery	0.27	-0.94	1.48
Hx of Percutaneous coronary intervention	0.49	-0.76	1.75
Substance or alcohol abuse	**-2.27**	**-3.92**	**-0.62**
Current smoker	-0.51	-1.70	0.67
Dementia	-2.80	-6.71	1.10
COPD	**-1.14**	**-2.15**	**-0.12**
Stroke	-2.10	-4.78	0.58
Depression	-1.13	-2.56	0.30
Arthritis	**-11.68**	**-13.60**	**-9.76**
Diabetes mellitus	-0.49	-1.55	0.57
Coronary revascularization during index admission	1.09	-0.17	2.35
Cardiogenic shock	-2.68	-8.31	2.95
Hypotensive episode	-0.09	-2.52	2.33
Positive stress test	-0.01	-2.44	2.43
Admitted to Tertiary VA	0.02	-1.37	1.40
ST-segment elevation MI on ECG	**1.88**	**0.64**	**3.12**
Mean serum creatinine (per 1 mg/dL increment)	-0.34	-1.00	0.32
Left bundle branch block on ECG	-1.87	-4.72	0.97
Cardiac catheterization during index admission	-0.40	-1.62	0.81
Discharge diagnosis Unstable Angina (vs. MI)	0.24	-0.97	1.46
Inverse Mills Ratio *			

* Inverse Mills Ratio: Variable derived from the Heckman Selection Equation; MI: myocardial infarction; Hx: History; COPD: chronic obstructive pulmonary disease; ECG: electrocardiogram

Variables significantly associated with better 7-month physical health status included ST-segment elevation MI on electrocardiogram and coronary revascularization during the index ACS hospital admission. Variables significantly associated with worse 7-month physical health status included older age, history of prior CABG surgery, chronic heart failure, arthritis, COPD, stroke, depression, diabetes, and elevated serum creatinine during index ACS admission.

Variables significantly associated with better 7-month mental health status included older age and ST-segment elevation MI on electrocardiogram. Variables significantly associated with worse 7-month mental health status included a history of prior MI, alcohol and/or substance abuse, COPD, and arthritis.

### Heckman selection model

The Heckman selection model (modeling response to the SF-36) is presented in Table 2. Older age, prior PCI, and history of COPD were associated with an increased likelihood of survey response, whereas a history of alcohol or substance abuse, depression, and have had a do not resuscitate order during their index hospitalization were associated with a decreased likelihood of survey response.

### Final PCS and MCS models

The final multivariable models for PCS and MCS (after addition of the IMR variables from the Heckman selection model) are presented in Tables 4 and 6. There was little evidence of selection bias for the PCS model. None of the results of inference testing for significance changed between the initial model and the model with the IMR variable added. Furthermore, the changes in magnitude of parameter estimates were not large, and the parameter estimate on the IMR variable itself was not significant.

**Table 6:** MCS Model Corrected For Response Bias (N = 1605)

		**95% Confidence Interval**	
**Variables**	**Coefficient**	**Lower Limit**	**Upper Limit**
Mean patient age (per 1 year increment)	**0.06**	**0.00**	**0.12**
Male gender	0.25	-2.65	3.15
Caucasian race	-0.51	-1.24	0.22
Hx of MI	**-1.24**	**-2.07**	**-0.40**
Hx of Chronic heart failure	0.17	-0.94	1.27
Hx of Coronary artery bypass graft surgery	-0.01	-1.21	1.18
Hx of Percutaneous coronary intervention	-0.19	-1.36	0.99
Substance or alcohol abuse	-1.55	-3.39	0.29
Current smoker	-0.17	-1.46	1.13
Dementia	-2.81	-6.90	1.27
COPD	**-1.76**	**-2.80**	**-0.72**
Stroke	-1.75	-4.25	0.75
Depression	-1.33	-2.71	0.04
Arthritis	**-10.80**	**-12.83**	**-8.78**
Diabetes mellitus	-0.35	-1.43	0.72
Coronary revascularization during index admission	0.70	-0.48	1.89
Cardiogenic shock	-2.58	-7.28	2.12
Hypotensive episode	-0.16	-2.26	1.94
Positive stress test	0.01	-2.24	2.25
Admitted to Tertiary VA	0.24	-1.10	1.58
ST-segment elevation MI on ECG	**1.92**	**0.91**	**2.92**
Mean serum creatinine (per 1 mg/dL increment)	-0.37	-0.97	0.24
Left bundle branch block on ECG	-2.00	-4.63	0.63
Cardiac catheterization during index admission	-0.46	-1.63	0.72
Discharge diagnosis Unstable Angina (vs. MI)	0.04	-1.23	1.32
Inverse Mills Ratio *	**-8.93**	**-10.73**	**-7.13**

* Inverse Mills Ratio: Variable derived from the Heckman Selection Equation; MI: myocardial infarction; Hx: History; COPD: chronic obstructive pulmonary disease; ECG: electrocardiogram

By contrast, when the IMR variable was inserted into the initial MCS model, we found evidence of selection bias. In this case, the parameter estimate for history of alcohol or substance abuse changed from significant to insignificant with the introduction of the IMR variable. Therefore, it appears that a history of alcohol or substance abuse was associated with lower likelihood of responding to the survey, but not directly associated with mental health status. In addition, a number of parameter estimates changed quantitatively, with larger changes than those observed in the PCS findings. Finally, the coefficient on the IMR variable itself was significant, and was negatively associated with MCS, implying that unobserved variables in the selection equation appear to be associated with worse MCS scores.

## Discussion

The goal of this study was to demonstrate the use of the Heckman two-step method to assess and correct for bias due to missing HRQL surveys in a clinical study of acute coronary syndrome patients. We created initial multivariable models of 7-month post-ACS physical and mental health status using data only from survey respondents. Then, using the Heckman method, we modeled survey non-response, derived an Inverse Mills Ratio variable for each patient that captured the likelihood of survey response, and incorporated this variable into our initial models to assess and correct for potential bias from survey non-response.

We found that our initial model of 7-month physical health status was not biased due to survey non-response. In contrast, our initial model of 7-month mental health status was biased. After controlling for bias due to non-response, a history of alcohol or substance abuse was no longer associated with mental health status. Furthermore, the magnitude of the parameter estimates for several of the other predictor variables in the MCS model changed after accounting for bias due to survey non-response.

Given these results, biased parameter estimates of the association between these variables and mental health status would have been reported if we had used the standard approach to evaluating the predictors of HRQL outcomes in this population. Furthermore, we might have concluded that alcohol/substance abuse was significantly associated with mental health status outcomes following ACS, and may therefore be an important target for interventions to improve post-ACS HRQL (e.g. improving alcohol screening and treatment). While alcohol/substance abuse may be important for other reasons, it would have been incorrect to conclude that it was associated with HRQL in our study population. Rather, it was a marker for survey non-response. This analysis demonstrates the utility of the Heckman method in its application for assessing and correcting survey response bias in clinical studies of HRQL.

Health-related quality of life data are usually not missing at random, and failure to account for missing HRQL assessments can bias estimates of associations and may lead to inappropriate conclusions about the determinants of HRQL outcomes [1-3]. Often, HRQL data are missing in systematic ways that can be estimated and controlled for. This study demonstrates the use of one technique to accomplish this, the Heckman two-step method [5-8]. To date, the Heckman method has rarely been utilized in studies reported in the medical literature, although it was previously used in one study assessing the impact of selection on medication use among older patients [7].

There are other statistical techniques, or approaches, to assess and correct for bias resulting from missing HRQL surveys, including index function models, propensity scores, instrumental variables, and multiple imputation methods [2-4,23]. The Heckman method is one example of an index function model. Generally, the index function approach to missing HRQL data is to model whether or not HRQL surveys were completed (i.e. the dependent variable is survey completion). This allows an estimation of the 'likelihood' that a given patient would complete a survey based on their clinical characteristics and/or other process or structure of care variables. This information, in turn, is used to assess and correct for bias in the primary model of interest (i.e. the model of quality of life outcome). Therefore, a primary strength of the Heckman method is that it not only permits the assessment of selection bias, it corrects for the bias, and does so in an informative way that may yield new insights into the association between patient characteristics or processes of care and outcomes of interest such as HRQL. In the Heckman method, the assumption is made that the error term in the outcome equation is standard normal, the distribution assumed in classical linear regression. Other index functions allow other distributional assumptions to be made for the error term in the outcome equation, such as logistic.

Propensity score approaches can be analogous to the Heckman method in that a multivariable model of survey non-response is developed and the probability of survey non-response is used to stratify the study population and/or the propensity score is used as an independent variable in the primary HRQL models. In other words, propensity scores are similar to the Heckman method in that the predicted probability of non-response is used as the basis for assessing the impact of missing data and controlling for it [23]. Unlike a propensity score, however, which is often entered directly into the outcome equation as a predictor, the non-linear transformation from the prediction into the IMR variable in the Heckman method is one of the safeguards against colinearity in the outcome equation.

Instrumental variable approaches are also used to address similar questions to those addressed by the Heckman method. In the full instrumental variable approach, a single exogenous variable (called the instrument) is used to stratify the full sample, removing the effect of the correlated error terms that lead to biased estimates [24]. An instrumental variable approach can be a very powerful approach to controlling selection bias. However, it can be very difficult to find an appropriate and adequate instrumental variable. The Heckman approach offers a more flexible, if less powerful, approach, and adds information about the underlying processes by which selection arose. It should be noted that propensity scores can be used as instrumental variables, when a suitable instrument is found.

Finally, multiple imputation methods can be employed to address missing HRQL data [2]. In contrast to the Heckman and other approaches described thus far, multiple imputation methods derive missing values from existing data in the dataset, thereby creating a 'complete' dataset and eliminating the need to drop patients from analysis. Imputation thereby eliminates bias from missing data per se (i.e. there is no longer missing data), but is highly dependent on the validity of deriving the missing HRQL survey data from the existing dataset. It is important to note that in this paper, we are focused on missing surveys rather than incomplete surveys (i.e. missing data elements within a survey). In this regard, multiple imputation will most often be employed in studies with serial measurements of HRQL over time, such that HRQL data before and/or after the time point of interest can inform the missing data imputation. The Heckman method can be used even for a single point in time, cross-sectional assessment of HRQL, as in our analysis in which we measured HRQL at only one time point.

The Heckman method has several limitations. First, the selection equation must have at least one variable that is associated with survey response but not the outcome of the study (i.e. HRQL). In some clinical applications, the inability to identify such a variable may make it difficult to use the full Heckman method to control bias. However, it is still possible to use the first step of estimating a selection equation to assess the degree to which selection bias may affect the parameter estimates in an outcome equation. If there are variables that are significant in both the selection equation and the outcomes equation, it is likely that there is bias due to selection effects in the outcome equation. Acknowledging this and commenting on the likely magnitude of effect may provide helpful guidance to clinicians and other researchers. Another limitation of the Heckman method is that this technique depends heavily on the quality of the data available for the selection equation. If the amount of variance explained is relatively low, then there is a possibility that selection bias in the outcomes equation may not be detected. In other words, the Heckman method can be under-powered for the detection of bias in some cases.

Finally, the Heckman method is very sensitive to how the model is specified; in other words, omitting variables that are associated with either non-response or with the outcome of interest (in this case, health related quality of life measures) can lead to inaccurate findings and biased estimates of the parameters in the final models. Careful attention to specifying the models, and ensuring that model specification follows what is known in the literature to be associated with the outcomes of interest is essential [4].

## Conclusions

This study demonstrated the use of the Heckman two-step method to assess and control for bias from missing HRQL surveys in a clinical study. We found that our mental health status model was significantly biased due to missing HRQL assessments. Recognition and correction of this bias changed the parameter estimates of association and the factors that we concluded were associated with HRQL seven months following hospital admission for an acute coronary syndrome. We conclude that the Heckman two-step method may be a valuable tool in the assessment and correction of selection bias in clinical studies of HRQL.

## Authors' contributions

AES conceived of the study, participated in design and coordination, conducted analyses, and drafted the manuscript; MEP conducted analyses and contributed to the manuscript; DJM and JAS reviewed and contributed to the manuscript; JSR participated in the design and coordination of the study and participated in the drafting and revision of the manuscript. All authors read and approved the final manuscript.
